# The Role of Conventional TACE (cTACE) and DEBIRI-TACE in Colorectal Cancer Liver Metastases

**DOI:** 10.3390/cancers14061503

**Published:** 2022-03-15

**Authors:** Thomas J. Vogl, Maximilian Lahrsow

**Affiliations:** Department of Diagnostic and Interventional Radiology, University Hospital Frankfurt, Theodor-Stern-Kai 7, 60590 Frankfurt am Main, Germany; t.vogl@em.uni-frankfurt.de

**Keywords:** colorectal cancer (CRC), colorectal cancer liver metastases (CRLM), conventional/traditional transarterial chemoembolization (cTACE), drug-eluting beads TACE (DEB-TACE), irinotecan-loaded drug-eluting beads TACE (DEBIRI-TACE), overall survival (OS), progression-free survival (PFS)

## Abstract

**Simple Summary:**

Liver metastases of colorectal cancer have an enormous clinical impact and prevalence. As colorectal cancer is one of the most common cancers, such patients are routinely encountered in day-to-day practice. Still, surgery and systemic chemotherapy constitute the first-line therapies depending on clinical setting. However, if patients present with non-resectable liver-only or liver-dominant metastases and/or do not respond to systemic chemotherapy, local therapies based on a vascular approach can be offered by interventional radiologists to achieve local tumor control or to downstage tumor burden. These therapies are generally called transarterial chemoembolization (TACE). Such treatments can also be combined with systemic or other local therapies. Depending on local practice and expertise, TACE can be offered with a combination of chemotherapeutic agents and embolizing agents or drug-eluting beads which embolize the metastases and its feeding vascular supply and release a chemotherapeutic agent over time. In the following review we compare these different approaches in the local therapy of liver metastases of colorectal cancer by presenting representative study results.

**Abstract:**

Colorectal cancer (CRC) is one of the most common tumor entities worldwide and a common cause of cancer-associated death. Colorectal cancer liver metastases (CRLM) thereby constitute a severe life-limiting factor. The therapy of CRLM presents a major challenge and surgical resection as well as systemic chemotherapy remain the first-line treatment options. Over the years several locoregional, vascular- and image-based treatments offered by interventional radiologists have emerged when conventional therapies fail, or metastases recurrence occurs. Among such options is the conventional/traditional transarterial chemoembolization (cTACE) by local injection of a combination of chemotherapeutic- and embolic-agents. A similar treatment is the more recent irinotecan-loaded drug-eluting beads TACE (DEBIRI-TACE), which are administered using the same approach. Numerous studies have shown that these different types of chemoembolization can be applied in different clinical settings safely. Furthermore, such treatments can also be combined with other local or systemic therapies. Unfortunately, due to the incoherent patient populations of studies investigating TACE in CRLM, critics state that the definite evidence supporting positive patient outcomes is still lacking. In the following article we review studies on conventional and DEBIRI-TACE. Although highly dependent on the clinical setting, prior therapies and generally the study population, cTACE and DEBIRI-TACE show comparable results. We present the most representative studies on the different chemoembolization procedures and compare the results. Although there is compelling evidence for both approaches, further studies are necessary to determine which patients profit most from these therapies. In conclusion, we determine TACE to be a viable option in CRLM in different clinical settings. Nevertheless, a multidisciplinary approach is desired to offer patients the best possible care.

## 1. Introduction

Colorectal cancer (CRC) is the third most common malignant entity and second most deadly cancer, causing 1.9 million incidence cases and claiming approximately 0.9 million lives worldwide in 2020 [1]. The most common location of metastases outside of lymph nodes is the liver [2]. At the time of diagnosis of the CRC, about fifteen percent of patients present with synchronous liver metastases, which is an independent poor prognostic factor [3]. Furthermore, at the time of diagnosis, up to a third of patients have already developed liver metastases that are occult to imaging [4,5]. Because of these tumor characteristics, colorectal cancer and its metastases are a common clinical encounter, always requiring an interdisciplinary approach by a tumor board. Surgical resection of colorectal cancer liver metastases (CRLM) is considered to be the only curative treatment option, but only about one in five patients are suitable candidates for surgical resection [6]. Patients with liver metastases of colorectal cancer only have a median survival of seven months when treated with the best supportive care [7]. Surgical resection of liver metastases results in a five-year survival rate of 37% and a 10-year survival rate of 22% [8]. Although modern therapies have historically improved the survival rates of patients with CRLM, patients without metastatic disease still have the best survival rates [9]. In general, systemic chemotherapy constitutes the backbone in the treatment of CRLM and can be used in many clinical settings. Neoadjuvant systemic chemotherapy can be applied to achieve a secondary respectability in patients that are not suitable for surgery by downsizing and -staging metastases. It can be used to improve progression-free survival rates and remission rates after surgical resection was performed and can also be applied in a palliative situation to maintain quality of life and to control local disease [10]. Still, the typical chemotherapeutic agents are applied in the following regimes: FOLFOX, which is a combination of 5-FU, leucovorin and oxaliplatin; and FOLFIRI, which is a combination of 5-FU, leucovorin and irinotecan [11]. Targeted/biological therapies that attack signal points of tumor growth have also been introduced and are applied in combination with classical chemotherapy [12]. For patients that fail to respond to conventional therapies or show tumor progression after treatment, several vascular-based therapies can be offered by interventional radiologists [13]. These vascular approaches are transarterial chemoembolizations (TACE) performed by introducing a catheter in the femoral artery and advancing it into metastases feeding arteries in the liver. There, a combination of chemotherapeutic and embolizing agents is injected. Usually, TACE is used in cases of heavy metastases burden in palliative situations to achieve disease management/control. But TACE can also be combined with other local therapies like percutaneous thermal ablation or systemic chemotherapy [14]. Historically, transarterial embolization started in the 1970s [15]. There are several TACE techniques available today. The original chemoembolization is called conventional TACE (cTACE), using a combination of chemotherapy and embolizing agents [16,17]. A similar, more recent technique is the injection of drug-eluting beads, called DEB-TACE or, more specifically, irinotecan-loaded drug-eluting beads TACE (DEBIRI-TACE). In general, TACE has been used in different tumor entities and is a standard therapy in the treatment of hepatocellular carcinoma (HCC). Studies on chemoembolization in CRLM lack comparability, as different techniques of TACE exist and the study populations are very heterogenous in terms of tumor burden and prior therapies. Principally, studies indicate that cTACE and DEB-TACE are somewhat equal in terms of safety and tumor control rates [18,19]. The following review will bring the representative studies on cTACE and DEB-TACE under scrutiny (Table 1).

## 2. Transarterial Approach

The basis of chemoembolization is the dual blood supply of the liver by the portal vein and the hepatic artery [20]. While the liver parenchyma is mainly supplied by the portal vein, liver metastases often receive their blood supply by branches of the hepatic artery [21]. DEBIRI-TACE and cTACE use the same vascular approach. The technical aspect of transarterial chemoembolization is performed in a traditional fashion. In transarterial therapies after local anaesthesia, the common femoral artery is punctured, and a sheath and catheter are introduced by using the Seldinger technique [22]. Via the hepatic artery, a catheter is advanced into the tumor or metastases that are feeding blood vessels. The selective approach allows for an intraarterial injection of chemotherapeutic agents, which is referred to transarterial chemoperfusion (TACP) and embolic material, which is known as transarterial embolization (TAE). By this technique, healthy liver parenchyma is mostly spared [7]. The combination of chemotherapeutics and embolic agents is called TACE [16]. The direct injection of chemotherapeutic agents into the tumor feeding arteries leads to a higher intratumoral concentration than systemic chemotherapy [23]. The embolizing agents cause a longer contact time of chemotherapeutics and tumor tissue, increasing the chemotherapeutic agent’s effect [24]. Exclusion criteria for treatment with chemoembolization are in general extrahepatic metastases, poor performance status, high tumor burden (more than 70% of liver parenchyma), high total bilirubin serum levels, reduced hepatic synthesis, renal dysfunction and complete portal vein thrombosis [25]. These general contraindications are somewhat dependent on the local practice. Studies often include patients with extrahepatic metastases, which leads to a reduced comparability with other studies. The most common side effect is the postembolization syndrome, consisting of fever, abdominal pain and nausea. Rare complications are liver abscess, cholecystitis, gall bladder necrosis, non-target embolization and transient bilirubin elevation [26,27]. Both DEB-TACE and cTACE have a similar toxicity profile, although studies indicate that DEB-TACE has fewer adverse events than conventional TACE [28]. The cause for this observation could be the lower peak concentration of chemotherapeutic agents in blood plasma during the treatment with drug-eluting beads, as beads release the chemotherapeutic agent over time [29]. At the same time local hepatic toxicity has been reported to be higher in DEB-TACE [30]. Patients undergoing transarterial chemoembolization can be treated in an outpatient setting or spend approximately one day in the hospital for observation after treatment. cTACE and DEB-TACE can be performed repeatedly and is often performed in four-week intervals [25,31,32]. In comparison to cTACE, with is typically performed in a selective or sub-selective fashion, DEBIRI-TACE is commonly administered in a lobar approach. Irinotecan is a prodrug which is converted into its active form by liver parenchyma [13].

## 3. cTACE

**Figure 1:**
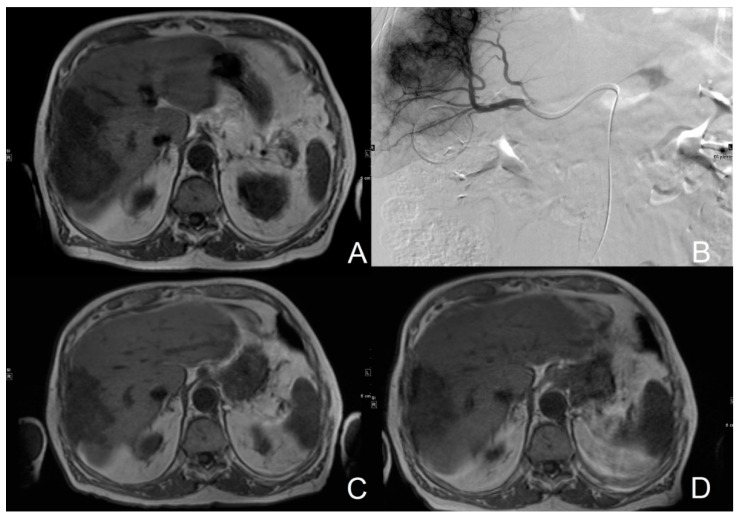
T1-weighted MR images of a 78-year old patient with non-resectable liver metastases of colorectal carcinoma prior to palliative cTACE (**A**). Angiogram during cTACE procedure shows liver metastases (**B**). Repetitive cTACE resulted in stabilization of disease (**C**). 20 months after initial chemoembolization, imaging still showed stabilization of disease (**D**).

There are several studies on conventional TACE, having evolved from hepatic artery infusion (HAI) and transarterial embolization (TAE). The combination of chemotherapeutic agents and embolic agents is still dependent on institutional preference and experience (Figure 1). The therapeutic regime used in TACE is also influenced by prior systemic chemotherapy. A large, retrospective study of 564 patients treated with cTACE showed a median survival of 14.3 months. Patients were treated with different chemotherapeutic agents and embolized with ethiodized oil and starch microspheres. The indication was a significant prognostic factor, and patients treated in a neoadjuvant setting fared best [25]. A more recent follow-up study by the same authors analyzed 452 patients that were treated with cTACE. All patients were embolized with a combination of ethiodized oil (Lipiodol^®^) and degradable starch microspheres (EmboCept^®^). Chemotherapeutic agents used were Mitomycin C, Gemcitabine, Irinotecan, and Cisplatin in different combinations depending on prior systemic chemotherapy. Approximately 60% of patients were treated with a triple combination of Mitomycin C, Irinotecan, and Cisplatin. The average number of cTACE sessions per patient was 5.9. Almost half of the patient population was treated palliatively while the other half was treated in a neoadjuvant setting followed by thermal ablation. Patients treated with a triple combination showed best treatment response. Patients undergoing neoadjuvant cTACE showed significantly longer overall survival (OS), (25.8 months vs. 12.6 months) and progression-free survival (PFS), (10.8 months vs. 5.9 months) when compared to the palliative cohort. In the study, the number and size of liver metastases were significant prognostic factors for overall and progression-free survival in the patient group treated neoadjuvantly [32]. The presence of extrahepatic metastasis proved to be significant factor for overall and progression-free survival in the neoadjuvant and palliative cohort. The authors concluded that cTACE is a feasible treatment option in advanced colorectal cancer liver metastases. In combination with further thermal ablation, survival rates are further improved. In another study, 24 patients were treated with cTACE with cisplatin powder and degradable starch following failure of FOLFOX systemic chemotherapy. This resulted in a median overall survival of 21.1 months and a median hepatic progression-free survival of 8.8 months. However, several patients were eligible for surgical resection of metastases after treatment, which might overestimate the overall survival benefit of the interventional therapy [33]. An investigation on 21 patients performed TACE with a mixture of mitomycin and degradable starch microspheres in a palliative setting, resulting in a median survival of 13.8 months and a progression-free survival of 5.8 months. Patients with extrahepatic metastases were also included [34]. Another study which included patients with extrahepatic metastases showed a median OS of 7.7 months. The rugs used were polyvinyl alcohol, cisplatin, doxorubicin and mitomycin [35]. A larger study with 121 patients that showed failure to systemic chemotherapy performed cTACE with cisplatin, doxorubicin, mitomycin C, ethiodized oil, and polyvinyl alcohol particles. Overall survival and progression-free survival were calculated at nine months and five months, respectively. Survival was significantly better when cTACE was performed prior to third line systemic chemotherapy [36]. In conclusion, these studies show that cTACE is a feasible treatment option in patients in which conventional treatment fails. It offers good local control rates in different clinical settings. Therefore, it seems evident that a combination of different chemotherapeutic agents is favorable in treatments. Transarterial chemoembolization is a proven treatment option, especially in combination with other interventional therapies like thermal ablation.

## 4. DEBIRI-TACE

Based on cTACE, drug-eluting beads TACE (called DEB-TACE) was introduced, which utilizes permanent microspheres that function as embolic agents and release chemotherapeutic agents over time. As compared to cTACE, DEB-TACE has a longer history in the management in hepatocellular carcinoma, although there is no clear evidence showing a definite advantage of one over the other [37]. In CRLM, irinotecan-loaded drug-eluting beads TACE (DEBIRI-TACE) is currently commonly used for the intra-arterial delivery of irinotecan. There are different types of beads available for loading with irinotecan. The most commonly used size of beads is in between 100 μm and 300 μm, although studies indicate that smaller beads might yield higher tumor response rates [38]. A multinational, multicentral study with 55 patients proved the efficacy and safety of DEBIRI-TACE in patients with colorectal cancer liver metastases that were refractory to systemic chemotherapy. On average, two DEBIRI sessions were performed in each patient. Overall survival was 19 months and progression-free survival was 11 months. These results might be confounded by the fact that thirty percent of patients received simultaneous systemic chemotherapy [39]. Evaluating the safety was also the aim of a prospective, double institutional phase II clinical study that included 82 patients. All patients had undergone at least two systemic therapies prior to TACE. On average, each patient underwent 2.2 chemoembolization procedures with tolerable side effects and toxicity. The DEBIRI therapy resulted in an OS of 25 months and a PFS of eight months. The authors concluded that DEB-TACE can be proposed as a palliative therapy in patients with unresectable CRLM that do not respond to systemic chemotherapy to achieve disease management/control [40]. A more recent, single institute study with 27 patients reported an overall survival of 5.4 months after treatment with microspheres preloaded with irinotecan. Forty percent of patients presented with extrahepatic metastases at the beginning of TACE therapy. Each patient had received on average 1.3 therapy sessions with DEB-TACE with beads of 100 μm to 300 μm in size. The shorter OS compared to prior studies was attributed to the heavily pretreated patient population and the presence of extrahepatic metastases in almost half of the population. Further analysis also revealed that patients with a better performance status and fewer prior lines of systemic chemotherapy showed longer overall survival. No correlation between the presence of extrahepatic metastases and shortened survival was reported, possibly due to the small study cohort [41]. DEB-TACE and systemic FOLFIRI therapy were directly compared in a study with 74 patients. The OS for DEBIRI was 22 months and for FOLFIRI it was 15 months. The PFS for DEBIR was seven months and FOLFIRI it was four months. These differences were calculated to be statistically significant, which indicated that transarterial therapy might be superior to systemic chemotherapy. Surprisingly, an improvement in time to progression of extrahepatic metastases was also observed in the DEBIRI cohort, although it did not reach statistical significance [42]. 

**Table 1:** Overview of studies on cTACE and DEB-TACE.

Table 452.	Patient Number	Overall Survival (Months)	Progression-Free Survival (Months)	Reference
**cTACE**				
palliative and neoadjuvant	452	12.6 (palliative) 25.8 (neoadjuvant)	5.9 (palliative) 10.8 (neoadjuvant)	[32]
cTACE after FOLFOX failure	24	21.1	8.8	[33]
palliative	21	13.8	5.8	[34]
palliative	21	7.7		[35]
palliative	121	9	5	[36]
**DEBIRI-TACE**				
efficacy	55	19	11	[39]
palliative/safety	82	25	8	[40]
palliative/safety	27	5.4		[41]
efficacy and safety	30 (17 TACE vs. 13 TACE and bevacizumab)	5.8 TACE vs. 12 TACE and bevacizumab	4 TACE vs. 6 TACE and bevacizumab	[43]
efficacy	57	37.4	10.8	[44]
efficacy	74	22 (DEBIRI-group)	7 (DEBIRI-group)	[42]
efficacy	70		15.3 (DEBIRI and FOLFOX/bevacizumab)	[45]
neoadjuvant	40	50.9		[46]

DEBIRI-TACE has also been investigated in combination with systemic chemotherapeutic agents and compared to transarterial treatment. In a study with a total of 30 patients, 17 patients were treated with TACE with irinotecan loaded polyethylene glycol embolics and 13 patients were treated with TACE with the same regime and followed by intravenous bevacizumab. The combination is believed to increase the antiangiogenic effect of both therapies. The study demonstrated that the combination of DEB-TACE and bevacizumab is well tolerated by patients. OS and PFS in the TACE cohort were 5.8 and four months. The combination of TACE followed by bevacizumab resulted in an OS of 12 months and a PFS of six months. The difference in survival rates between the two cohorts was statistically significant. The combination therapy showed similar survival results compared to prior studies that focused on just TACE treatment. In contrast to this, the TACE-only cohort in the study showed below average survival rates. A possible explanation might be that the whole patient population was heavily pretreated, with half of all patients having received more than two systemic chemotherapies. In the end, due to the small study population, this could not be concluded definitively [43]. 

Another interesting, prospective and multi-center study with 57 patients evaluated patients with unresectable CRLM that were naïve to systemic chemotherapy. A combination of transarterial DEBIRI and systemic FOLFOX was used. Each DEBIRI-TACE treatment was performed two to three days after a systemic chemotherapy cycle. Four chemoembolizations per patient were performed in a sequential unilobar approach, or two chemoembolizations in a bilobar approach. In a planned safety analysis after 27 patient cases, it was determined to treat the rest of the patients with the sequential unilobar approach due to decreased toxicity. The OS was reported at 37.4 months and the PFS at 10.8 months. Thirty-three percent of patients underwent secondary resection or ablation therapy after treatment with DEBIRI and FOLFOX. This cohort showed a progression-free survival of 13 months, while median overall survival was not reached. Although high response rates were demonstrated, the authors did not recommend DEBIRI and FOLFOX as primary treatments for CRLM as the initially hypothesized nine months of PFS were not reached. The occurrence of post-embolization syndrome appeared somewhat high, which might have been due to a cumulative effect of local and systemic therapy and the chemotherapy-naïve patients. On the other hand, one in three patients reached secondary resectability after treatment. In the end, the authors concluded that further studies are required to find a place of a combination of DEBIRI and FOLFOX in the complex treatment of CRLM [44]. 

Another comparison study between systemic chemotherapy and a combination of systemic therapy and DEBIRI-TACE has also been performed in a multi-center trial. Seventy patients with unresectable CRLM and liver dominant disease that were naïve to systemic chemotherapy were either assigned to a group that received DEBIRI-TACE and systemic FOLFOX or to a group that received systemic FOLFOX. The additional use of bevacizumab in both groups was left at the discretion of the treating medical oncologist. DEBIRI was performed in a lobar approach in two sessions, and the average number of sessions was four per patient. Irinotecan was loaded on a spherical hydrogel device. The patient group treated with transarterial and systemic chemotherapy showed a statistically significant improvement in response rates compared to the FOLFOX group, without an increase of systemic toxicity or side effects. Furthermore, progression-free survival was also longer in the combination group (15.2 months vs. 7.6 months), and more patients in the combination group became eligible to hepatic resection of metastases. What is also notable is that in the DEBIRI/FOLFOX arm of the study that showed prolonged survival rates, more patients with extrahepatic metastases and more patients with a poorer performance status were included [45]. The two aforementioned studies indicate that the ideal timing of local and systemic therapy as well as the number of DEBIRI sessions have yet to be determined.

Limited data is available on using DEBIRI-TACE in a neoadjuvant setting. A study with 40 patients receiving TACE with drug eluting PVA microspheres loaded with irinotecan one month prior to surgical resection of metastases examined survival rates. It should be noted that all patients were initially eligible for resection of CRLM. Each patient received one DEBIRI session with a median interval between TACE and surgery of 30 days. No patient received systemic chemotherapy following surgical resection. This resulted in an overall survival of 50.9 months, which was deemed comparable to systemic neoadjuvant chemotherapy followed by surgery. DEBIRI-TACE did not cause delay in surgical therapy and was well tolerated by patients with minimal systemic toxicity. Additionally, most of the resected metastatic lesions showed histologic major or complete pathologic response, comparable to a situation after multiple cycles of systemic chemotherapy. Importantly, local therapy with TACE did not negatively impact resectability of metastases or surgical outcome [46]. Such a neoadjuvant approach should also be investigated in combination with other systemic, antitumoral agents.

## 5. Conclusions

Since the initial catheter-based, transarterial hepatic therapies of the 1970s, TACE has evolved into a common treatment option offered by interventional radiology in different tumor entities. While TACE procedures started out as (and still are an established) therapy in HCC, chemoembolization has been extended to other hepatic malignancies, especially in cases of liver metastases. Among these, liver metastases of colorectal cancer play an enormous clinical role due to the high incidence of colorectal cancer [47]. Transarterial chemoembolization can be offered like systemic chemotherapy under several clinical circumstances, for example most commonly in a setting after the failure of systemic chemotherapy to achieve disease management/control, or neoadjuvantly and curatively prior to further thermal ablation or hepatic resection [26,48]. Traditionally/conventionally, TACE has been performed with a combination of one or more chemotherapeutic agent(s) and embolic materials of different kinds. Besides this so-called cTACE, in recent years the drug-eluting beads known as TACE have been developed, based on embolic microspheres that release irinotecan over time (DEBIRI-TACE). The use of one TACE procedure over the other in CRLM is commonly based on the local experience and preference of the interventional radiology department. Our review showed that there are many studies on the interventional treatment of liver metastases. A significant drawback of studies on TACE is the general small volume of the patient cohort. To the author’s knowledge, no head to head comparison between cTACE and DEBIRI-TACE in CRLM exists to date. There are also no robust studies comparing results between interventional therapies or alternative therapies. Furthermore, there is only preliminary evidence that addresses the combination of multiple therapies. From reviewing the available studies, it becomes evident that while often in a similar range, rates of overall survival and progression-free survival after cTACE and DEB-TACE are not comparable to each other. The reason for this is the heterogenous cohorts of patients in the available trials. Study populations present with different lines of prior chemotherapy and undergo TACE in different fashions (for example, regarding chemotherapeutic and embolic agent(s) in cTACE and types of beads in DEB-TACE). The absence or existence of extrahepatic metastases and the possible hepatic resection or ablation after chemoembolization are also constant confounders when comparing survival rates of TACE trials. The evidence suggests that both TACE procedures are feasible treatment options to manage or control disease after failure of systemic chemotherapy. There are compelling studies that also demonstrate a benefit in survival if cTACE is combined with further thermal ablation in cases of unresectable CRLM. National Comprehensive Cancer Network (NCCN) and European Society of Medical Oncology (ESMO) guidelines establish a role of vascular-based therapies in patients with liver-dominant metastases who failed systemic chemotherapy. Furthermore, guidelines recommend using such therapies in a (neo)adjuvant setting only in clinical trials [49,50]. The combination of chemotherapeutic agents and embolic agents is highly dependent on institutional preference, thereby reducing comparability between studies further. In several studies, therapeutic regime used in TACE was adjusted to prior applied systemic chemotherapy. In contrast to cTACE, DEBIRI-TACE has been combined in several studies with systemic chemotherapy, resulting in good response rates and prolonged survival. Further studies should increasingly focus on combining DEBIRI-TACE with systemic agents that block angiogenesis without increasing systemic toxicity. It appears logical that the combination of local and systemic therapy would be beneficial to achieve local tumor control and at the same time prevent or stabilize extrahepatic metastases. Future studies should incorporate the effects of combined therapies on distant metastases outside the liver. More and more studies also focus on biomarkers to predict treatment response to chemoembolization. The identification of such predictors could result in the early termination of an ineffective treatment. In the therapy with cTACE, diffusion-weighted imaging by measuring ADC values showed a promising predictive value in a small study population [51]. In a study with DEBIRI-TACE, serum CEA and CA 19.9 values were correlated with tumor response and survival. Although statistical significance was not met, further studies with more patients might reach a significant level [52]. Another potential biomarker for predicting treatment response after DEB-TACE is VEGFR1. Decreased VEGFR1 levels were noted after treatment, which is associated with an antitumoral effect and might signal a better prognosis. In the end, the prognostic value of VEGFR1 could not be confirmed conclusively, and further studies are required [53]. Besides cTACE and DEBIRI-TACE, there is Y-90 transarterial radioembolization (TARE), which is also being conducted via a transarterial approach. TARE also shows promising survival rates in patients with CRLM and has also been combined with systemic chemotherapy [54,55]. As in other vascular approaches, the ideal patient population eligible for TARE still has to be determined [56]. In a recent investigation, prognostic factors were identified that provided a good correlation with survival rates after radioembolization of CRLM. As in TACE, the size of metastases and the presence of extrahepatic metastases highly influenced survival [57]. Research should be conducted comparing TACE and TARE, although it can be hypothesized that future studies will also lack comparability due to heterogenous patient cohorts.

In conclusion, the heterogeneity of patients and the number of different therapies available demonstrate that, regardless of interventional radiology expertise, a multidisciplinary approach with tumor boards including (among others) surgeons and medical oncologists is obligatory when encountering patients with liver metastases of colorectal cancer. Generally speaking, the discussed interventional therapies are not widely adopted and are usually reserved for specialized oncological and often academic centers. Currently, no general applicable strategy can be sketched regarding the combination of interventional therapies and other treatments.
